# De Novo Transcriptome Analysis Reveals Flowering-Related Genes That Potentially Contribute to Flowering-Time Control in the Japanese Cultivated Gentian *Gentiana triflora*

**DOI:** 10.3390/ijms231911754

**Published:** 2022-10-04

**Authors:** Tomoyuki Takase, Motoki Shimizu, Shigekazu Takahashi, Keiichirou Nemoto, Fumina Goto, Chiharu Yoshida, Akira Abe, Masahiro Nishihara

**Affiliations:** Iwate Biotechnology Research Center, Kitakami 024-0003, Japan

**Keywords:** *B-BOX* genes, CONSTANS, day-neutral plant, flowering, FT, Japanese gentians, *MADS*-*box* genes, photoperiod, RNA-seq

## Abstract

Japanese cultivated gentians are perennial plants that flower in early summer to late autumn in Japan, depending on the cultivar. Several flowering-related genes, including *GtFT1* and *GtTFL1*, are known to be involved in regulating flowering time, but many such genes remain unidentified. In this study, we obtained transcriptome profiling data using the *Gentiana triflora* cultivar ‘Maciry’, which typically flowers in late July. We conducted deep RNA sequencing analysis using gentian plants grown under natural field conditions for three months before flowering. To investigate diurnal changes, the plants were sampled at 4 h intervals over 24 h. Using these transcriptome data, we determined the expression profiles of leaves based on homology searches against the Flowering-Interactive Database of *Arabidopsis*. In particular, we focused on transcription factor genes, belonging to the *BBX* and *MADS-box* families, and analyzed their developmental and diurnal variation. The expression levels of representative *BBX* genes were also analyzed under long- and short-day conditions using in-vitro-grown seedlings, and the expression patterns of some *BBX* genes differed. Clustering analysis revealed that the transcription factor genes were coexpressed with *GtFT1*. Overall, these expression profiles will facilitate further analysis of the molecular mechanisms underlying the control of flowering time in gentians.

## 1. Introduction

Japanese cultivated gentians (*Gentiana triflora*, *Gentiana scabra*, and their hybrids) are the flowers most frequently used to decorate graves on special occasions, such as the Bon Festival, ‘Obon’, in mid-August and Equinox week, ‘Ohigan,’ in late September [1]. Therefore, the flowering time of gentians is an important factor for farmers seeking to ship the flowers to market in a timely manner. Except for a minority of potted plants grown in greenhouses, gentians are usually cultivated in the field; thus, controlling their growth and flowering time is challenging. Several pre- and post-harvest studies have been conducted to assess the quality and longevity of cut gentian flowers [2,3,4], and the results depend on cultivar and species; therefore, a universal method for extending gentian flower life has not been established. In contrast to chrysanthemums, which are short-day plants for which flowering time can be controlled using photoperiodic lighting (e.g., by day-length extension or night breaks) [5], Japanese cultivated gentians are considered day-neutral plants [6], like tomato and cucumber [7,8]. Therefore, managing gentian flowering time using light is relatively difficult, and most farmers instead cultivate several cultivars with different flowering times, thereby accounting for the year-by-year fluctuations in flowering time in the field. However, this strategy is wasteful, and gentian flower farmers would prefer to use a validated flowering-time control method or cultivars insusceptible to yearly weather conditions.

The molecular mechanisms underlying flowering in Japanese gentians have been investigated in several studies. For example, Imamura et al. [9] isolated and characterized three members of the *FLOWERING LOCUS T* (*FT*)/*TERMINAL FLOWER 1* (*TFL1*) gene family in *G. triflora*. Their analyses suggested that gentian flowering time is related to the expression levels of two homologous genes, *GtFT1* and *GtTFL1*. FT and TFL1 are known to have antagonistic functions in the floral initiation of many crops, i.e., FT activates the flowering pathway, whereas TFL1 represses flowering [10]. In addition, *MADS-box SHORT VEGETATIVE PHASE*-*like* genes (*GtSVP-L1* and *GtSVP-L2*) have been cloned and characterized in *G. triflora* [11]. According to virus-induced gene silencing analysis, GtSVP-L1 acts as a negative regulator of flowering [11]. Of the *MADS-box* genes known to act in flowering-time regulation and floral organ identity, 14 genes belonging to A–E classes have been cloned from *G. scabra*, and functional analyses have been conducted mainly on the regulation of flower shape [12,13].

Japanese cultivated gentians are perennial plants, and the overwintering process affects the timing of bud break in spring and indirectly affects the timing of flowering in summer and autumn. Takahashi et al. [14] found that gentiobiose, a gentian-specific oligosaccharide, acts as a signal that releases overwintering buds from dormancy through the AsA–GSH cycle. The role of *GtFT2*, a homolog of *GtFT1*, in dormancy regulation was also revealed by Takahashi et al. [15]. They found that *GtFT2* is involved in the release from endodormancy, functioning as an accelerator. Additionally, the gentian orthologs of *FRUITFULL* (*GtFUL*) and *GtSVP-L1* seemingly act downstream of *GtFT2* to prevent untimely budbreak during ecodormancy. Takahashi et al. [15] cloned several flowering-related genes, including *FLOWERING LOCUS C* (*GtFLC-L*), *SUPPRESSOR OF OVEREXPRESSION OF CO 1* (*GtSOC1a* and *GtSOC1b*), and *LEAFY* (*GtLFY*), and they analyzed the seasonal expression levels of these genes, although their floral initiation-related functions remained unidentified.

Although several genes related to phase transition, including flowering and overwintering, have been cloned and analyzed in Japanese gentians, more studies are necessary to achieve a full understanding of the molecular mechanisms underlying flowering in these plants and to facilitate the control of flowering time. For example, pioneering studies on *Arabidopsis* have revealed that the *CONSTANS* (*CO*) gene plays a central role in the photoperiod response; therefore, *CO* and *CO-LIKE* (*COL*) genes have been cloned and analyzed in many crops, including cereals, vegetables, fruits, and flowers [16,17,18,19,20]. *CO* and *COL* genes are also involved in various phase transition events in plants. For example, CO is involved in photoperiodic tuberization in potato [21] as well as growth cessation and bud set in poplar and bud set and bud burst in Norway spruce [22,23]. Notably, in tomato, a day-neutral plant, *SlCO*/*SlCOL* genes have been cloned and analyzed in relation to photoperiodic signaling and flowering; three genes, namely *SlCOL*, *SlCOL4a*, and *SlCOL4b*, were speculated to control tomato flowering through their interaction with *SFT* [24]. However, studies on cloning and expression analyses of *CO*/*COL* genes in gentians are lacking. Several *MADS-box* genes are known to be involved in flowering control in higher plants, but gentian *MADS-box* genes have been studied exclusively in relation to overwintering [15] and determining floral organ identity [12,13]; thus, the involvement of the *MADS-box* gene family in Japanese gentian flowering warrants further investigation. As Japanese cultivated gentians are day-neutral plants, their floral initiation is not dependent on photoperiod requirements; however, it is unclear whether *CO*/*COL* genes, associated with the photoperiod response, are conserved in gentians and affect flowering.

RNA sequencing (RNA-seq) is a powerful next-generation sequencing technique used to perform transcriptome analysis. It has advantages over microarray technology and has been applied extensively in plant-based research [25,26], including in several studies of *Gentiana* species. For example, in *Gentiana macrophylla*, RNA-seq analysis was used to identify the genes involved in secoiridoid biosynthesis [27,28]. In two other gentian species, *Gentiana rigescens* and *Gentiana straminea,* RNA-seq analysis was used to determine the genes involved in the biosynthesis of active ingredients [29,30]. We also applied RNA-seq analysis to Japanese gentians in relation to flower color and flower opening [31,32,33]. However, RNA-seq analysis has yet to be targeted at flowering in *Gentiana* species.

Thus, the aim of the present study was to produce gene catalogs and expression profiles of the developmental and diurnal changes in flowering-related genes in a gentian species. To this end, field-grown *G. triflora* plants were the subjects of RNA-seq analysis for three months prior to flowering. To evaluate the usefulness of the RNA-seq-based gene catalog, we first analyzed *B-BOX* (*BBX)* family genes, including the *CO/COL* family, and *MADS-box* genes, as this gene family is known to regulate flowering in other plant species. Next, using in-vitro-grown seedlings, we tested the photoperiodic response in selected *BBX* genes, confirming that some of these genes have photoperiodic responses under different conditions. Subsequently, we identified the transcription factor genes coexpressed with *GtFT1* via clustering analysis, finding genes that were likely related to flowering and plant growth. Overall, this study revealed candidate genes that may be involved in the regulation of flowering time in gentians. These data will facilitate further research on flowering in gentians at the molecular level and improve our understanding of the molecular mechanisms underlying the control of flowering time in these plants, which will enable the development of molecular markers and breeding of suitable cultivars for flowering control.

## 2. Results

### 2.1. Identification of Reference Genes Expressed in Gentian Leaves, and Gene Annotation via BLAST Analysis and Protein Motif Analysis

**Figure 1:**
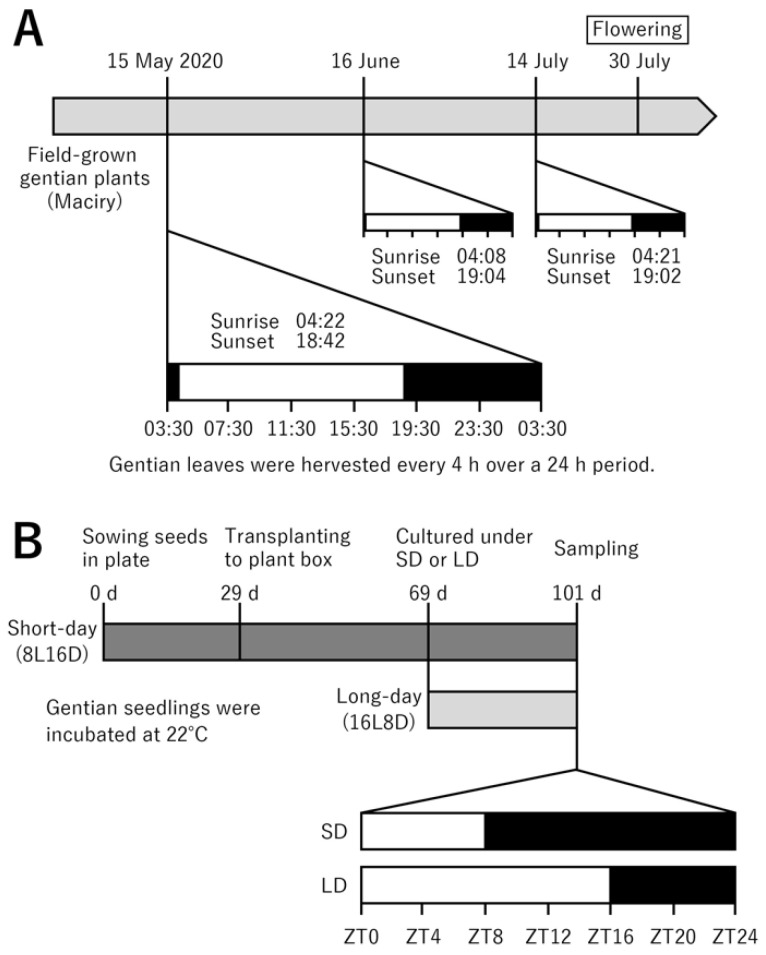
Schematic diagram of the sampling of field- and in-vitro-cultured gentians. (**A**) Field-grown gentian ‘Maciry’ (3-year-old) plants were used. The third to fourth fully opened leaves were sampled at seven time points and subjected to RNA-seq analysis. (**B**) Aseptic seedlings cultured in plant boxes under long- and short-day conditions were used. The third and fourth leaves from the top were sampled at seven time points and subjected to qRT-PCR analysis. White and black boxes indicate light and dark periods, respectively.

The leaves of field-grown gentian plants were sampled as shown in Figure 1A. A list of the bulks used for RNA-seq is shown in Supplementary Table S1. The filtered RNA-seq reads were assembled de novo using Trinity [34], with 521,292 transcripts detected. The coding regions of the assembled transcripts were predicted using TransDecoder [35]. Subsequently, Cluster Database at High Identity with Tolerance (CD-HIT) [36] was used to reduce transcript redundancy and obtain unique genes. Finally, 37,919 transcripts were obtained. BUSCO analysis was performed to evaluate the completeness of the assembly against a dataset set of 1614 core genes in Embryophyta [37]; the completeness of the transcript set was 87.3%, which is comparable to 89.0% in our previous RNA-seq analysis of corolla [33]. RNA-seq results are summarized in Table 1. The transcripts were annotated using a local BLASTX search for *Arabidopsis thaliana* (downloaded from ftp.ensemblgenomes.org/pub/plants/release-42/fasta/arabidopsis_thaliana/pep/ (accessed on 1 September 2022)) and the UniProt (downloaded from ftp.ebi.ac.uk/pub/databases/uniprot/current_release/knowledgebase/complete/ (accessed on 1 September 2022)) protein database. Gene expression levels were calculated from the transcripts per kilobase million (TPM) values using the RSEM software within the Trinity package. The expression levels of all contigs and the annotation results are shown in Supplementary Table S2.

### 2.2. Expression Profiling of Flowering-Related Genes in Leaves over a Three-Month Period Prior to Flowering

**Figure 2:**
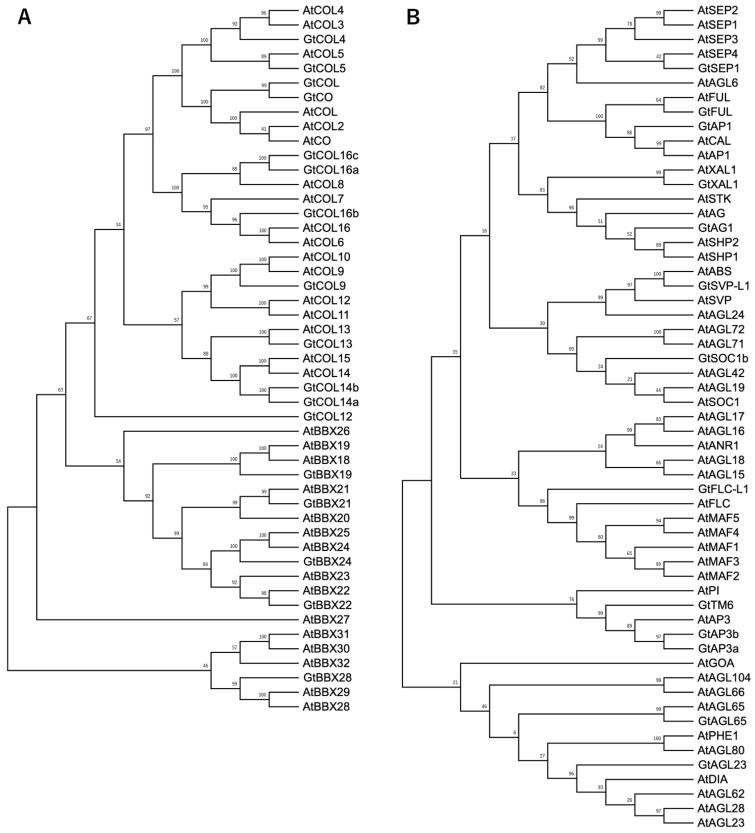
Phylogenetic analysis of BBX and MADS-box proteins in gentians and *Arabidopsis* *thaliana*. (**A**) Phylogenic tree of BBX family proteins in gentians and *A. thaliana*. (**B**) Phylogenic tree of MADS-box family proteins in gentians and *A. thaliana*.

We searched contigs known to be flowering-related genes in *Arabidopsis* using the Flowering-Interactive Database (FLOR-ID; http://www.phytosystems.ulg.ac.be/florid/ (accessed on 10 March 2021)) [38]. Among the genes present in FLOR-ID, our RNA-seq contigs had 212 hits (65.2%; Table 2 and Table S3). This gene catalog covered 44.2–74.1% of FLOR-ID genes depending on the pathway. First, we analyzed the gene expression patterns of *BBX* and *MADS*-*box* family genes. A list of these genes is provided in Table S4. Seventeen *BBX* and thirteen *MADS-box* genes were included, and phylogenetic analysis using *Arabidopsis* genes indicated that gentian genes were distributed in most clades (Figure 2A,B). The conserved motifs in the *BBX* genes are shown in Figure S1; the assembled contigs were full length, except for *GtCOL12*.

**Figure 3:**
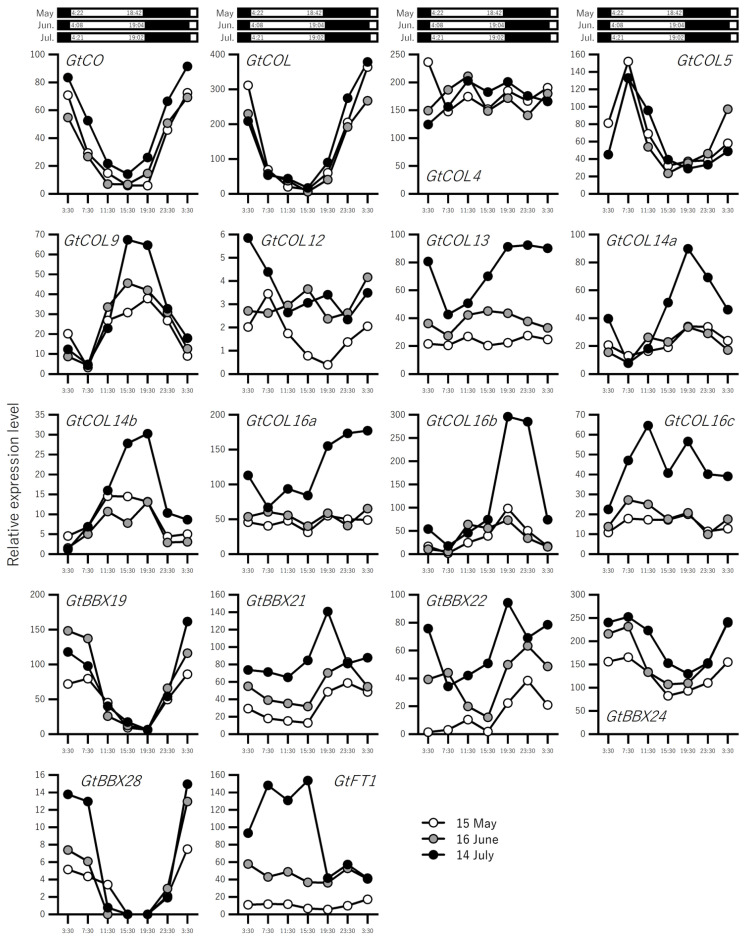
Expression profiles of *BBX* genes in field-grown gentians. Gentian plants grown under natural conditions in the field were used. The details of sampling are shown in Figure 1A. White and black boxes indicate day and night periods, respectively.

**Figure 4:**
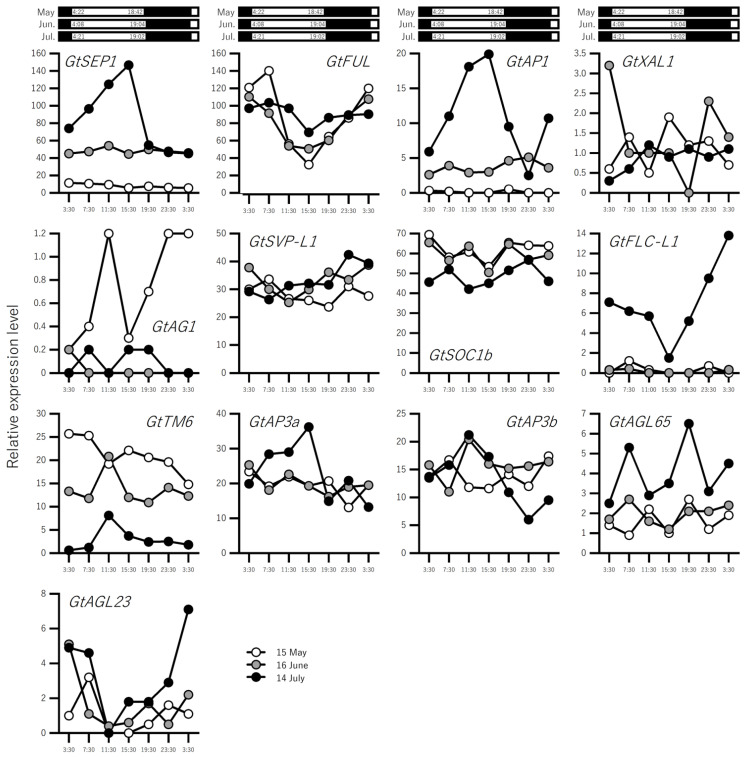
Expression profiles of *MADS-box* genes in field-grown gentians. Gentian plants grown under natural conditions in the field were used. The details of sampling are shown in Figure 1A. White and black boxes indicate day and night periods, respectively.

The expression profiles of the gentian *BBX* and *MADS*-box genes are shown in Figure 3 and Figure 4, respectively. We performed real-time reverse transcription-PCR (qRT-PCR) to validate the expression levels (TPM) obtained via RNA-seq analysis. For this purpose, we searched for internal reference genes for the standardization of gene expression because the expression of the ubiquitin gene used previously fluctuated in samples collected on 14 July (Figure S2). To select these reference genes, we used the following criteria: TPM values at all points were >100, and the value of each point was 0.67–1.50 times the average of all points. Using these criteria, three genes were found to be stably expressed in all samples: *MALE GAMETOPHYTE DEFECTIVE 1* (*GtMGP1*), *20S PROTEASOME ALPHA SUBUNIT PAD1* (*GtPAD1*), and *GLYCERALDEHYDE-3-PHOSPHATE DEHYDROGENASE C SUBUNIT 1* (*GtGAPC1*) (Figure S3). Because the variation in *GtMGP1* gene expression was the lowest among these genes, we selected *GtMGP1* as an internal standard. We subjected *GtFT1* and four *BBX* genes near to *Arabidopsis CO*, i.e., *GtCO*, *GtCOL*, *GtCOL4*, and *GtCOL5*, to qRT-PCR, finding that the expression profile patterns were similar according to RNA-seq (Figure 3) and qRT-PCR (Figure S2) analyses. Therefore, the TPM values obtained via RNA-seq were considered reliable and suitable for detailed expression profiling analysis.

*GtFT1* expression increased from May to June, but clear variation in expression within one day was not observed. In contrast, *GtFT1* expression levels increased markedly during the day in July (Figure 3 and Figure S2). *GtFT2*, involved in the phase transition of overwintering buds, was not detected in our RNA-seq analysis. Seventeen *BBX* genes showed gene-dependent developmental and diurnal changes, but no genes showed similar expression patterns to those of *GtFT1* (Figure 3). The expression patterns of 13 *MADS-box* genes are shown in Figure 4. The expression profiles were also gene-dependent, but the expression patterns of *SEPALLATA*1 (*GtSEP1*) and *APETALA1* (*GtAP1*) were similar to that of *GtFT1*, whereas the expression patterns of *GtSVP-L1* and *GtFLC-L1* differed from those of *GtFT1*. The expression levels of *GtFUL* increased slightly during development, whereas those of *GtSOC1b* decreased.

### 2.3. Effect of Photoperiod on the Expression of BBX Genes in In Vitro–Grown Seedlings

**Figure 5:**
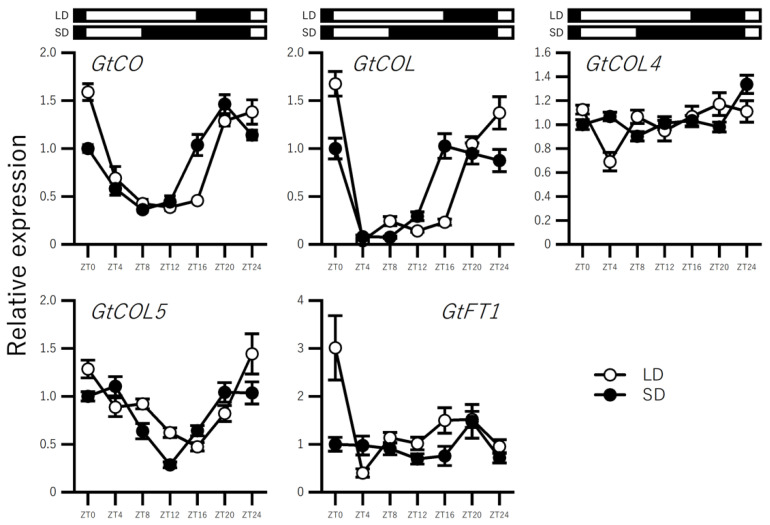
qRT-PCR analysis of representative gentian *BBX* genes and *GtFT1* in in-vitro-cultured plants. Seedlings grown under long- and short-day conditions for 32 days were used. The details of sampling are shown in Figure 1B. The Y-axis shows relative expression levels, which are shown with the short day, ZT0, value as 1. The X-axis shows time points. White and black boxes indicate light and dark periods, respectively.

We further investigated gentian *BBX* genes by analyzing the effects of different day lengths on their expression levels. It was difficult to control the day-length conditions in the field-grown gentians; therefore, we used three-month-old in-vitro-grown gentian seedlings (Figure 1B). Flowering was not observed in these in-vitro-grown gentians (Figure S4). As expected, qRT-PCR analysis revealed that *GtFT1* expression was not induced under either day-length condition, i.e., either 16 h light/8 h dark or 8 h light/16 h dark (Figure 5). The expression profiles of representative *BBX* genes, including *GtCO*, *GtCOL*, *GtCOL4*, and *GtCOL5*, over 24 h are shown in Figure 5. *GtCO*, *GtCOL*, and *GtCOL5* showed different expression patterns (repressed in the light) under long- and short-day conditions, whereas the expression patterns of *GtCOL4* barely changed over 24 h. Thus, some *BBX* genes exhibited altered expression levels in response to day length, even when *GtFT1* expression was not induced.

### 2.4. Coexpression Cluster Analysis

**Figure 6:**
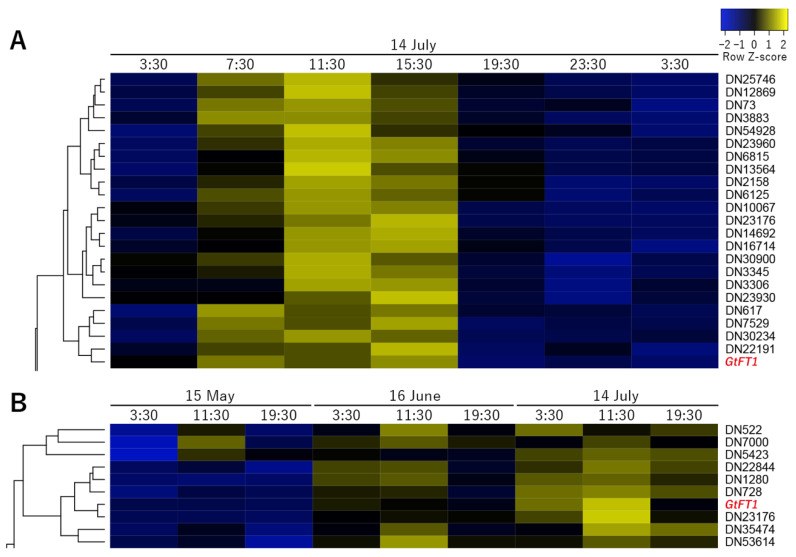
Coexpression cluster analysis of transcription factor genes coexpressed with *GtFT1.* (**A**) Clustering analysis was performed using the TPM values from 14 July, as described in the Materials and Methods. (**B**) Clustering analysis was performed using the TPM values from three months, as described in the Materials and Methods.

Because *GtFT1* expression levels in July showed diurnal changes compared with those in May and June (Figure 3 and Figure S3), we searched for the genes coexpressed with *GtFT1* in July using coexpression analysis. To this end, transcription factor genes were selected among all contigs, and only genes with specific expression levels (TPM values of ≥1 at any point) were used. The list of genes used is shown in Table S5, and a heatmap including all 1095 contigs used is shown in Figure S5. The genes belonging to the same clade as *GtFT1* are shown in Table 3 and in closeup in Figure 6A. The list includes *MADS-box* genes, *GtSEP1*, *GtAP3a*, and *GtAP3b* but does not include any *BBX* genes. The clade included a *CRYPTOCHROME-INTERACTING basic helix-loop-helix 1* (*CIB1*) homolog, known to be a positive regulator of *FT* expression. Notably, several hormone signaling-related genes were also included in the gene list. The expression profiles of phytohormone-related genes found in Table 2 are also shown in Figure S7.

To identify the genes coexpressed with *GtFT1* during development over three months, clustering analysis of transcription factor genes was performed using data from the three-month period. A heatmap of all genes is shown in Figure S6, and a list of all identified genes is shown in Table S6. Genes belonging to the *GtFT1* clade are shown in Table 4 and in closeup in Figure 6B. The clade included *GtBBX22,* a homolog of *AtBBX22* involved in light and phytohormone pathways, as well as a *MADS-box* gene, *GtSEP1*, which was also observed in the coexpression analysis of the July data.

## 3. Discussion

We performed transcriptome analysis of field-grown Japanese cultivated gentians using the typical cultivar ‘Maciry,’ with data collected over three months prior to flowering and over 24 h at 4 h intervals (Table S1). De novo assembly resulted in 37,919 contigs which included 212 FLOR-ID homologous genes (Table 2). Gene expression levels prior to floral initiation and with diurnal changes were obtained (Table S2), and the reliability of the results was validated by qRT-PCR analysis (Figure S5). The obtained data improve our understanding of the molecular mechanisms underlying flowering in gentians; in particular, we obtained analysis results for the *BBX* and *MADS-box* gene families, which are well-known for their roles in the flower development process.

We identified 17 *BBX* genes belonging to group I–V (Figure 2A and Figure S5). In other plant species, ca. 30 *BBX* genes are usually identified as B-box protein family genes (e.g., 32 in *Arabidopsis*, 29 in tomato, and 30 in rice) [39]. Therefore, more genes are likely to be present in gentians. The unidentified genes might be expressed in other organs or at other times, such as under stress-induced conditions, e.g., temperature stress, water stress, and pathogen attack. However, the *CO/COL* genes expressed in leaves are generally important for photoperiodic flowering control [40], and expression profiling data are useful for identifying the candidate genes involved in flowering control. The expression of some *BBX* genes was induced on 14 July (about 2 weeks prior to flowering) with the expression of *GtFT1*, but the expression patterns of the *BBX* genes did not match that of *GtFT1* (Figure 3). Notably, *GtCO* and *GtCOL*, homologs of *Arabidopsis CO* and *COL*, showed similar expression patterns over three months, i.e., the expression levels decreased under light and increased under dark in the three-month period. Although the expression patterns of *GtCOL5* did not change over three months, the expression of this gene was induced in the morning when the induction of *GtFT1* expression began (07:30), suggesting that *GtCOL5* rather than *GtCO* and *GtCOL* contributes to the induction of *GtFT1* expression. It is possible that repressor genes were expressed in both May and June to suppress *GtFT1* induction or that other cooperative genes are lacking, even when *GtCOL5* is expressed. It may also be possible that the protein stability of GtCOL5 is involved in the activation of *GtFT1* transcription as reported in *Arabidopsis* CO [41].

Because it was difficult to subject field-grown gentians to day-length treatments, we analyzed the photoperiod responses of in-vitro-grown seedlings (Figure 1B). We found that three *BBX* genes, namely *GtCO*, *GtCOL*, and *GtCOL5*, were expressed at different levels in response to day length (Figure 5). As expected, *GtFT1* expression was not fully induced at the young seedling stage as the plants were not ready for flowering; nevertheless, the photoperiodic response of the *BBX* genes suggested their involvement in flowering control in response to different day lengths. However, the gene expression levels of these genes in field-grown gentians did not change over three months, reflecting the similar day lengths on 15 May (14 h 20 m), 16 June (14 h 56 m), and 14 July (14 h 41 m). Therefore, another study is necessary to reveal the responsiveness of these genes to photoperiod in mature field-grown gentians, which could be achieved using artificial shading or light supplementation. In a previous study, *SlCOL*, *SlCOL4a*, and *SlCOL4b*, among the 13 members in the *CO/COL* family, were identified as potential candidate activators through their interactions with *SFT* in day-neutral tomato plants [24]. These tomato genes had different diurnal rhythms under long- and short-day conditions, and they belonged to group I, like *Arabidopsis* CO, COL, and COL5, which function as flowering inducers. Whether gentian *CO/COL* family genes also regulate flowering in response to photoperiodic conditions remains unknown but warrants further study.

Thirteen *MADS-box* genes were identified via RNA-seq (Figure 3B and Table S4). In previous studies focusing on the identification of genes that regulate floral organ identity in *G. scabra* [12,13], 14 *MADS-box* genes belonging to A–E classes were identified, some of which were expressed at low levels in leaves. In our RNA-seq, 7 of these 14 genes were identified, indicating that these genes were expressed to some extent in leaves. The expression patterns of *GtAP1, GtAP3a*, and *GtSEP1* (closest to *AtSEP4)* were found to be similar to that of *GtFT1* (Figure 4). *Arabidopsis AP1, AP3*, and *SEP* genes expressed in the floral meristem are known as identity genes that specify floral organs and meristem identity [42,43]. *GtAP1, GtAP3a*, and *GtSEP1* are also expressed in floral organs as A, B, and E class genes, respectively [12]. Thus, it is likely that these homolog genes play the same roles in gentians. However, their expression was not limited to flowers, and the TPM value of *GtSEP1* was >100 on 14 July (maximum value: 146.73 at 15:30), suggesting the existence of any functional roles in leaves. *Arabidopsis SEP1–3* genes are expressed specifically in inflorescence tissues, whereas *SEP4* (also known as *AGL3*) is also expressed in leaves and floral stems [44], although functional analysis of SEP4 in relation to flowering time was not conducted. In *Isatis indigotica*, the silencing of *IiSEP4*, a homolog of *Arabidopsis SEP4*, delayed flowering time, whereas the overexpression of *IiSEP4* in *Arabidopsis* led to early flowering in addition to changes in floral organs [45,46]. Thus, further detailed analysis is necessary to gain insights into the involvement of GtSEP1 in gentian flowering control. Takahashi et al. [15] identified *GtAP1*, *GtFUL*, and *GtFLC-L* in *G. triflora,* and these genes were among our RNA-seq contigs (Table S4) However, some genes, such as *TFL1* and *LFY* homologs, were not among our RNA-seq contigs (Table S4), indicating that genes expressed predominantly in the shoot apical meristem (SAM) were missing. Thus, to complete the transcriptome analysis in gentians, RNA-seq should be performed for other organs, including flowers, SAMs, and overwintering buds. We are preparing these samples with the aim of obtaining comprehensive transcriptome data for gentians.

According to coexpression cluster analysis of our RNA-seq data, 23 genes were coexpressed with *GtFT1* on 14 July over 24 h (Table 3), including several flowering-related genes. *CIB1*, which is involved in *CRY2*-dependent regulation of flowering, was one of these genes. CIB1 and CO act together to regulate *FT* transcription and flowering [47]; thus, the *CIB1* homolog in gentians likely has a similar function, although the interacting *BBX* genes remain to be identified. Floral homeotic genes, e.g., *GtAP3a*, *GtAP3b*, and *GtSEP1*, were also coexpressed, and these may regulate flowering time as well as floral organ identity (as discussed above). Ten genes were detected in our coexpression cluster analysis of data from the three-month period (Table 4), and these genes are considered candidates for the regulation of flowering time as well as plant development in gentians. The gene lists shown in Table 3 and Table 4 also included several plant hormone-related genes involved in auxin, abscisic acid, and ethylene signaling. As shown in Figure S7, gene expression levels of gibberellin metabolism enzyme were not remarkably altered during development over three months, although some showed diurnal changes. Instead, the expression of one GA signaling pathway-related gene, *GtPIF4* (*PHYTOCHROME-INTERACTING FACTOR 4*), increased and showed reverse diurnal expression in July compared with May and June. *Arabidopsis PIF4* was revealed to bind the *FT* promoter and activate *FT* expression in cooperation with CO under warm ambient temperature [48]; therefore, GtPIF4 may also be involved in gentian flowering induction in July when the temperature rises. In contrast, *GtSPL3* expression was clearly reduced in July, but *Arabidopsis* SPL3 was reported to act as the photoperiodic activator signal in the FT-FD module in flowering [49]. It is likely that GtSPL3 may have the opposite function in gentian flowering, although further analyses are necessary. Some other phytohormone-related genes showed developmental and diurnal changes; therefore, these genes are also candidates along with the *BBX* and *MADS*-*box* genes mentioned above.

Efficient genome-editing and viral vectors have been established in Japanese gentians [50,51]; hence, in time, it will be possible to analyze these candidate genes. In addition, association analysis of gentian cultivars/lines with various flowering times would improve our understanding of the involvement of these genes in flowering-time control. Therefore, we are analyzing the DNA polymorphisms and expression levels of the candidate genes to determine their contribution to flowering time in Japanese cultivated gentians.

The first reference gentian genome, that of *Gentiana dahurica*, was constructed using three technologies (Nanopore, Illumina paired-end, and Hi-C) and has a total size of around 1.5 Gb [52]. In contrast, the Japanese cultivated gentians *G. triflora* and *G. scabra* have about 3-fold larger genome sizes, i.e., ca. 5 Gb, calculated according to their nuclear DNA contents [53]. Thus, the genome assembly of Japanese cultivated gentians will be more difficult, even using the latest technologies. Nevertheless, we are currently constructing a draft genome sequence of Japanese gentians because this sequence will be required to achieve a complete understanding of the various gene functions, especially those involved in flowering, in these plants.

## 4. Materials and Methods

### 4.1. Plant Materials

*Gentianatriflora* ‘Maciry,’ bred in Iwate prefecture, was used in this study. This cultivar is an F_1_ hybrid between two *G. triflora* breeding lines, and its flowering time is late July to early August depending on the year. The flowering time in 2020 was 30 July. Three-year-old plants were sampled in 2020. On 15 May, 16 June, and 14 July 2020, the fourth to fifth fully unfolded leaves from the top were collected from 10 independent plants for 24 h at 7 time points: 03:30, 07:30, 11:30, 15:30, 19:30, 23:30, 03:30. The middle parts of the leaves (ca. 100 mg) were excised, and the mid ribs were cut using scissors and placed into 2 mL screw cup tubes with beads. Five to ten samples were collected at each time point.

### 4.2. RNA-Seq Analysis

Total RNAs were isolated from the leaves using an RBC Total RNA Extraction Kit Mini (Plant) (SciTrove, Tokyo, Japan) according to the manufacturer’s instructions. DNase solution (Deoxyribonuclease, Nippon Gene, Tokyo, Japan; Buffer RDD, QIAGEN, Hilden, Germany) was used to conduct on-column digestion of genomic DNA. Construction of the cDNA libraries, sequencing, assembly, and functional annotation were performed as described previously [33]. The RNA-seq data was deposited in the DNA Data Bank of Japan (DDBJ; accession no. DRA014495). A summary of the generated RNA-seq data and the accession numbers deposited in DDBJ is shown in Table S1. During the quality control step, we filtered and discarded reads shorter than 50 bases and those with an average read quality of <20 and trimmed the poly A and adapter sequences using FaQCs version 2.08 [54].

### 4.3. Validation via qRT-PCR Analysis

The total RNAs isolated as described above were used for cDNA synthesis. qRT-PCR was performed using a QuantStudio 3 system (Thermo Fisher Scientific, Waltham, MA, USA) with Luna Universal qPCR Maser Mix (New England Biolabs, Ipswich, MA, USA). Reference genes for field-grown gentians were selected from the RNA-seq data, and the gentian *MGP1* gene was used as an internal control. The relative expression of genes was calculated using the 2^−ΔΔCt^ method. Primers are listed in Table S7.

### 4.4. Phylogenetic Analysis of the BBX and MADS-Box Family Proteins

BBX and MADS-box family proteins from gentians and *Arabidopsis* were used for phylogenic tree analysis. Protein sequences from *Arabidopsis* were obtained from the database of the National Center for Biotechnology Information (http://www.ncbi.nlm.nih.gov (accessed on 10 June 2022)). Maximum-likelihood phylogenic trees were constructed using MEGA X software (http://www.megasoftware.net/home (accessed on 1 September 2022)) with the JTT + G + I model and 1000 bootstrap replications.

### 4.5. Analysis of BBX Gene Expression in Response to Photoperiod in In-Vitro-Grown Seedlings

Seeds of ‘Maciry’ were aseptically sown on a plastic plate containing half-strength Murashige Skoog medium supplemented with 3% sucrose and 0.24% gellan gum at 22 °C for 29 days under short-day (8 h light/16 h dark) conditions (35 µmol m^−2^ s^−1^). Germinated seedlings were transferred to a plant box (5 shoots per box) and further cultured in a growth chamber for 40 days under the same short-day conditions. The seedlings were then cultured under short-day or long-day (16 h light/8 h dark) conditions (35 µmol m^−2^ s^−1^). After 32 days of incubation, the third to fourth leaves from the top were sampled at 4 h intervals over 24 h and stored at −80 °C until their use. All cultures were performed in a plant growth chamber (CLE-305, TOMY, Tokyo, Japan). The isolation of total RNAs and qRT-PCR analysis were performed as described above. Primers are listed in Table S7.

### 4.6. Gene Coexpression Analysis in Field-Grown Gentian Plants

To identify the genes coexpressed with *GtFT1* during developmental changes and diurnal changes, we performed clustering analysis. Specifically, we targeted transcription factor genes using the Plant Transcription Factor Database ver. 5.0 (http://planttfdb.gao-lab.org (accessed on 1 September 2022) [55,56,57,58]. Contigs with TPM values of >1.0 at any point were used. To select genes during the development stage, we used datasets from three time points (03:30, 11:30, and 19:30) on 15 May, 16 June, and 14 July (nine time points in total). For the analysis of diurnal change, seven time points (24 h) on 14 July were used when *GtFT1* was highly expressed. Hierarchical cluster analysis was performed based on the TPM value of each gene using Pearson correlation with an average-linkage clustering method via online tools (http://www.heatmapper.ca/expression/ (accessed on 28 June 2022)) [59].

## 5. Conclusions

To reveal the molecular mechanisms underlying flowering control, it is a prerequisite to identify the genes expressed during the developmental stages up to flowering as well as their responses to a variety of environmental conditions. In this study, we produced gene catalogs and performed expression profiling of field-grown gentians using RNA-seq. We successfully demonstrated the usefulness of our gene catalog by analyzing the expression profiles of *BBX* and *MADS-box* genes, which involved the screening of candidate genes involved in flowering. Coexpression cluster analysis also revealed several transcription factor genes potentially involved in flowering and growth regulation. Overall, this study represents the first step toward unraveling the molecular mechanisms underlying flowering-time and photoperiodic control in gentians. Flowering control is a highly complex process; therefore, the molecular and functional characterization of each candidate gene is warranted in future studies.

## Figures and Tables

**Table 1:** Outline of the results of RNA-seq analysis.

Trinity Status		
Contigs	Total number (>179 bp)	521,292
	Total assembled bases (bp)	411,975,909
	Average of contig length (bp)	790
	N50 (bp)	1360
Unigenes	Total number	37,919
	Total assembled bases (bp)	39,296,556
	Average of contig length (bp)	1036
	N50 (bp)	1206

**Table 2:** Summary of FLOR-ID genes found in gentian RNA-seq.

Pathway	No. of *Arabidopsis* FLOR-ID Genes	No. of Hit Genes
Photoperiod	139	82
Vernalization	90	63
Aging	53	26
Hormones	77	34
Ambient temperature	38	20
Sugar	46	21
Autonomous	116	86
Circadian clock	25	18
Total ^1^	325	212

^1^ Total numbers do not match, because some genes belong to several pathways.

**Table 3:** Coexpressed transcription factor genes with *GtFT1* over 24 h on 14 July.

Transcript ID	Gene Model	BlastP	Arabidopsis	Gentian
TRINITY_DN25746_c0_g1_i1.p1	AT5G22290.1	NAC domain-containing protein 89	*NAC089*	
TRINITY_DN12869_c1_g1_i7.p1	AT4G34530.1	Transcription factor bHLH63	*CIB1*	
TRINITY_DN73_c4_g1_i3.p1	AT2G16770.1	Basic leucine zipper 23	*bZIP23*	
TRINITY_DN3883_c0_g3_i1.p1	AT5G48150.3	Scarecrow-like transcription factor PAT1	*PAT1*	
TRINITY_DN54928_c0_g1_i3.p1	AT1G29280.1	Probable WRKY transcription factor 65	*WRKY65*	
TRINITY_DN23960_c0_g1_i5.p1	AT2G28550.3	Ethylene-responsive transcription factor RAP2-7	*TOE1*	
TRINITY_DN6815_c0_g2_i1.p1	AT2G44940.1	Ethylene-responsive transcription factor ERF034		
TRINITY_DN13564_c0_g1_i1.p1	AT2G28350.1	Auxin response factor 10	*ARF10*	
TRINITY_DN2158_c0_g1_i8.p1	AT3G61150.1	Homeobox-leucine zipper protein HDG1	*HDG1*	
TRINITY_DN6125_c0_g1_i1.p1	AT5G60850.1	Dof zinc finger protein DOF5.4	*OBP4*	
TRINITY_DN10067_c0_g2_i3.p1	AT4G06598.1	Uncharacterized protein At4g06598		
TRINITY_DN23176_c0_g1_i1.p1	AT2G03710.2	Agamous-like MADS-box protein AGL3	*SEP4*	*GtSEP1*
TRINITY_DN14692_c0_g1_i4.p1	AT1G74650.1		*MYB31*	
TRINITY_DN16714_c0_g1_i3.p1	AT3G11450.1			
TRINITY_DN30900_c0_g1_i2.p1	AT3G54340.1	Floral homeotic protein APETALA 3	*AP3*	*GtAP3b*
TRINITY_DN3345_c0_g3_i1.p1	AT3G23690.1	Transcription factor bHLH77	*CIL2*	
TRINITY_DN3306_c0_g1_i10.p1	AT2G30590.1	Probable WRKY transcription factor 21	*WRKY21*	
TRINITY_DN23930_c0_g2_i8.p1	AT3G53310.1	B3 domain-containing protein REM20		
TRINITY_DN617_c0_g2_i3.p1	AT3G10760.1			
TRINITY_DN7529_c0_g1_i1.p1	AT4G34590.1	bZIP transcription factor 11	*ATB2*	
TRINITY_DN30234_c0_g1_i2.p1	AT5G26930.1	GATA transcription factor 23	*GATA23*	
TRINITY_DN22191_c0_g1_i5.p1	AT3G54340.1	Floral homeotic protein APETALA 3	*AP3*	*GtAP3a*
TRINITY_DN2269_c0_g4_i2.p1	AT1G65480.2	Protein FLOWERING LOCUS T	*FT*	*GtFT1*

**Table 4:** Coexpressed transcription factor genes with *GtFT1* during development over three months.

Transcript ID	Gene Model	BlastP	Arabidopsis	Gentian
TRINITY_DN522_c0_g1_i1.p1	AT1G14920.1	DELLA protein GAI	*GAI*	
TRINITY_DN7000_c1_g1_i1.p1	AT4G00870.1	Transcription factor bHLH14		
TRINITY_DN5423_c0_g1_i2.p1	AT3G19860.1	Transcription factor bHLH121	*bHLH121*	
TRINITY_DN22844_c0_g1_i6.p1	AT5G01310.1		*APTX*	
TRINITY_DN1280_c1_g1_i2.p1	AT1G58110.1			
TRINITY_DN728_c3_g1_i2.p1	AT1G78600.1	B-box zinc finger protein 22	*BBX22*	*GtBBX22*
TRINITY_DN2269_c0_g4_i2.p1	AT1G65480.2	Protein FLOWERING LOCUS T	*FT*	*GtFT1*
TRINITY_DN23176_c0_g1_i1.p1	AT2G03710.2	Agamous-like MADS-box protein AGL3	*SEP4*	*GtSEP1*
TRINITY_DN35474_c0_g1_i1.p1	AT5G51980.2			
TRINITY_DN53614_c0_g1_i5.p1	AT1G67310.1	Calmodulin-binding transcription activator 4		

## Data Availability

All data supporting the findings of this study are available within the article and within its supplementary data published online. The raw RNA-seq data in this paper were deposited in DDBJ Sequence Read Archive (DRA) under the accession number DRA014495.

## Supplementary Materials

The following supporting information can be downloaded at: https://www.mdpi.com/article/10.3390/ijms231911754/s1.

## References

[1] Kodama K., The Japanese Society of Hortuculture Science (2006). IV-9, Gentian. Horticulture in Japan 2006.

[2] Eason J.R., Morgan E.R., Mullan A.C., Burge G.K. (2004). Display life of *Gentiana* flowers is cultivar specific and influenced by sucrose, gibberellin, fluoride, and postharvest storage. N. Z. J. Crop Hortic. Sci..

[3] Shimizu-Yumoto H., Ichimura K. (2012). Effects of ethylene, pollination, and ethylene inhibitor treatments on flower senescence of gentians. Postharvest Biol. Technol..

[4] Çelikel F.G., Rybczyński J.J., Davey M.R., Mikuła A. (2015). Postharvest physiology of flowers from the family Gentianaceae. The Gentianaceae—Volume 2: Biotechnology and Applications.

[5] Higuchi Y., Narumi T., Oda A., Nakano Y., Sumitomo K., Fukai S., Hisamatsu T. (2013). The gated induction system of a systemic floral inhibitor, antiflorigen, determines obligate short-day flowering in chrysanthemums. Proc. Natl. Acad. Sci. USA.

[6] Hisamatsu T., Hisamatsu T. (2014). Denshosaibai no Kiso to Jissen.

[7] Song J., Zhang S., Wang X., Sun S., Liu Z., Wang K., Wan H., Zhou G., Li R., Yu H. (2020). Variations in both *FTL1* and *SP5G*, two tomato FT paralogs, control day-neutral flowering. Mol. Plant.

[8] Yang A., Xu Q., Hong Z., Wang X., Zeng K., Yan L., Liu Y., Zhu Z., Wang H., Xu Y. (2022). Modified photoperiod response of CsFT promotes day neutrality and early flowering in cultivated cucumber. Theor. Appl. Genet..

[9] Imamura T., Nakatsuka T., Higuchi A., Nishihara M., Takahashi H. (2011). The gentian orthologs of the *FT*/*TFL1* gene family control floral initiation in *Gentiana*. Plant Cell Physiol..

[10] Higuchi Y. (2018). Florigen and anti-florigen: Flowering regulation in horticultural crops. Breed. Sci..

[11] Yamagishi N., Kume K., Hikage T., Takahashi Y., Bidadi H., Wakameda K., Saitoh Y., Yoshikawa N., Tsutsumi K. (2016). Identification and functional analysis of SVP ortholog in herbaceous perennial plant *Gentiana triflora*: Implication for its multifunctional roles. Plant Sci..

[12] Nakatsuka T., Saito M., Yamada E., Fujita K., Yamagishi N., Yoshikawa N., Nishihara M. (2015). Isolation and characterization of the C-class *MADS-box* gene involved in the formation of double flowers in Japanese gentian. BMC Plant Biol..

[13] Nakatsuka T., Saito M., Nishihara M. (2016). Functional characterization of duplicated B-class MADS-box genes in Japanese gentian. Plant Cell Rep..

[14] Takahashi H., Imamura T., Konno N., Takeda T., Fujita K., Konishi T., Nishihara M., Uchimiya H. (2014). The gentio-oligosaccharide gentiobiose functions in the modulation of bud dormancy in the herbaceous perennial *Gentiana*. Plant Cell.

[15] Takahashi H., Nishihara M., Yoshida C., Itoh K. (2022). Gentian *FLOWERING LOCUS T* orthologs regulate phase transitions: Floral induction and endodormancy release. Plant Physiol..

[16] Jin Q., Yin S., Li G., Guo T., Wan M., Li H., Li J., Ge X., King G.J., Li Z. (2021). Functional homoeologous alleles of *CONSTANS* contribute to seasonal crop type in rapeseed. Theor. Appl. Genet..

[17] Shalmani A., Fan S., Jia P., Li G., Muhammad I., Li Y., Sharif R., Dong F., Zuo X., Li K. (2018). Genome identification of B-BOX gene family members in seven *Rosaceae* species and their expression analysis in response to flower induction in *Malus domestica*. Molecules.

[18] Lu J., Sun J., Jiang A., Bai M., Fan C., Liu J., Ning G., Wang C. (2020). Alternate expression of *CONSTANS*-*LIKE 4* in short days and *CONSTANS* in long days facilitates day-neutral response in *Rosa chinensis*. J. Exp. Bot..

[19] Yang S., Weers B.D., Morishige D.T., Mullet J.E. (2014). *CONSTANS* is a photoperiod regulated activator of flowering in sorghum. BMC Plant Biol..

[20] Zhou R., Liu P., Li D., Zhang X., Wei X. (2018). Photoperiod response-related gene *SiCOL1* contributes to flowering in sesame. BMC Plant Biol..

[21] González-Schain N.D., Díaz-Mendoza M., Zurczak M., Suárez-López P. (2012). Potato CONSTANS is involved in photoperiodic tuberization in a graft-transmissible manner. Plant J..

[22] Chiang C., Viejo M., Aas O.T., Hobrak K.T., Strømme C.B., Fløistad I.S., Olsen J.E. (2021). Interactive effects of light quality during day extension and temperature on bud set, bud burst and *PaFTL2*, *PaCOL1-2* and *PaSOC1* expression in Norway spruce (*Picea abies* (L.) Karst.). Forests.

[23] Böhlenius H., Huang T., Charbonnel-Campaa L., Brunner A.M., Jansson S., Strauss S.H., Nilsson O. (2006). CO/*FT* regulatory module controls timing of flowering and seasonal growth cessation in trees. Science.

[24] Yang T., He Y., Niu S., Yan S., Zhang Y. (2020). Identification and characterization of the *CONSTANS* (*CO*)/*CONSTANS-like* (*COL*) genes related to photoperiodic signaling and flowering in tomato. Plant Sci..

[25] Martin L.B.B., Fei Z., Giovannoni J.J., Rose J.K.C. (2013). Catalyzing plant science research with RNA-seq. Front. Plant Sci..

[26] Sripathi V.R., Anche V.C., Gossett Z.B., Walker L.T., Louis I.V.-S. (2021). Recent applications of RNA sequencing in food and agriculture. Applications of RNA-Seq in Biology and Medicine.

[27] Hua W., Zheng P., He Y., Cui L., Kong W., Wang Z. (2014). An insight into the genes involved in secoiridoid biosynthesis in *Gentiana macrophylla* by RNA-seq. Mol. Biol. Rep..

[28] Cao X., Guo X., Yang X., Wang H., Hua W., He Y., Kang J., Wang Z. (2016). Transcriptional responses and gentiopicroside biosynthesis in methyl jasmonate-treated *Gentiana macrophylla* seedlings. PLoS ONE.

[29] Zhang X., Allan A.C., Li C., Wang Y., Yao Q. (2015). *De novo* assembly and characterization of the transcriptome of the Chinese medicinal herb, *Gentiana rigescens*. Int. J. Mol. Sci..

[30] Zhou D., Gao S., Wang H., Lei T., Shen J., Gao J., Chen S., Yin J., Liu J. (2016). De novo sequencing transcriptome of endemic *Gentiana straminea* (Gentianaceae) to identify genes involved in the biosynthesis of active ingredients. Gene.

[31] Sasaki N., Nemoto K., Nishizaki Y., Sugimoto N., Tasaki K., Watanabe A., Goto F., Higuchi A., Morgan E., Hikage T. (2021). Identification and characterization of xanthone biosynthetic genes contributing to the vivid red coloration of red-flowered gentian. Plant J..

[32] Tasaki K., Watanabe A., Nemoto K., Takahashi S., Goto F., Sasaki N., Hikage T., Nishihara M. (2022). Identification of candidate genes responsible for flower colour intensity in *Gentiana triflora*. Front. Plant Sci..

[33] Nemoto K., Niinae T., Goto F., Sugiyama N., Watanabe A., Shimizu M., Shiratake K., Nishihara M. (2022). Calcium-dependent protein kinase 16 phosphorylates and activates the aquaporin PIP2;2 to regulate reversible flower opening in *Gentiana scabra*. Plant Cell.

[34] Grabherr M.G., Haas B.J., Yassour M., Levin J.Z., Thompson D.A., Amit I., Adiconis X., Fan L., Raychowdhury R., Zeng Q. (2011). Full-length transcriptome assembly from RNA-Seq data without a reference genome. Nat. Biotechnol..

[35] Haas B.J., Papanicolaou A., Yassour M., Grabherr M., Blood P.D., Bowden J., Couger M.B., Eccles D., Li B., Lieber M. (2013). *De novo* transcript sequence reconstruction from RNA-seq using the Trinity platform for reference generation and analysis. Nat. Protoc..

[36] Fu L., Niu B., Zhu Z., Wu S., Li W. (2012). CD-HIT: Accelerated for clustering the next-generation sequencing data. Bioinformatics.

[37] Simão F.A., Waterhouse R.M., Ioannidis P., Kriventseva E.V., Zdobnov E.M. (2015). BUSCO: Assessing genome assembly and annotation completeness with single-copy orthologs. Bioinformatics.

[38] Bouché F., Lobet G., Tocquin P., Périlleux C. (2016). FLOR-ID: An interactive database of flowering-time gene networks in *Arabidopsis thaliana*. Nucleic Acids Res..

[39] Talar U., Kiełbowicz-Matuk A. (2021). Beyond Arabidopsis: BBX regulators in crop plants. Int. J. Mol. Sci..

[40] Valverde F. (2011). CONSTANS and the evolutionary origin of photoperiodic timing of flowering. J. Exp. Bot..

[41] Song Y.H., Estrada D.A., Johnson R.S., Kim S.K., Lee S.Y., MacCoss M.J., Imaizumi T. (2014). Distinct roles of FKF1, GIGANTEA, and ZEITLUPE proteins in the regulation of CONSTANS stability in Arabidopsis photoperiodic flowering. Proc. Natl. Acad. Sci. USA.

[42] Irish V.F. (2010). The flowering of Arabidopsis flower development. Plant J..

[43] Ditta G., Pinyopich A., Robles P., Pelaz S., Yanofsky M.F. (2004). The *SEP4* gene of *Arabidopsis thaliana* functions in floral organ and meristem identity. Curr. Biol..

[44] Huang H., Tudor M., Weiss C.A., Hu Y., Ma H. (1995). The *Arabidopsis* MADS-box gene *AGL3* is widely expressed and encodes a sequence-specific DNA-binding protein. Plant Mol. Biol..

[45] Ma Y.Q., Pu Z.Q., Tan X.M., Meng Q., Zhang K.L., Yang L., Ma Y.Y., Huang X., Xu Z.Q. (2022). *SEPALLATA*-like genes of *Isatis indigotica* can affect the architecture of the inflorescences and the development of the floral organs. PeerJ.

[46] Pu Z.Q., Ma Y.Y., Lu M.X., Ma Y.Q., Xu Z.Q. (2020). Cloning of a *SEPALLATA4*-like gene (*IiSEP4*) in *Isatis indigotica* Fortune and characterization of its function in *Arabidopsis thaliana*. Plant Physiol. Biochem..

[47] Liu Y., Li X., Ma D., Chen Z., Wang J.W., Liu H. (2018). CIB1 and CO interact to mediate CRY2-dependent regulation of flowering. EMBO Rep..

[48] Fernández V., Takahashi Y., Le Gourrierec J., Coupland G. (2016). Photoperiodic and thermosensory pathways interact through CONSTANS to promote flowering at high temperature under short days. Plant J..

[49] Jung J.H., Lee H.J., Ryu J.Y., Park C.M. (2016). SPL3/4/5 Integrate developmental aging and photoperiodic signals into the FT-FD module in *Arabidopsis* flowering. Mol. Plant.

[50] Nishihara M., Tasaki K., Sasaki N., Takahashi H. (2018). Development of basic technologies for improvement of breeding and cultivation of Japanese gentian. Breed. Sci..

[51] Fekih R., Yamagishi N., Yoshikawa N. (2016). Apple latent spherical virus vector-induced flowering for shortening the juvenile phase in Japanese gentian and lisianthus plants. Planta.

[52] Li T., Yu X., Ren Y., Kang M., Yang W., Feng L., Hu Q. (2022). The chromosome-level genome assembly of *Gentiana dahurica* (Gentianaceae) provides insights into gentiopicroside biosynthesis. DNA Res..

[53] Mishiba K., Yamane K., Nakatsuka T., Nakano Y., Yamamura S., Abe J., Kawamura H., Takahata Y., Nishihara M. (2009). Genetic relationships in the genus *Gentiana* based on chloroplast DNA sequence data and nuclear DNA content. Breed. Sci..

[54] Lo C.C., Chain P.S. (2014). Rapid evaluation and quality control of next generation sequencing data with FaQCs. BMC Bioinform..

[55] Tian F., Yang D.C., Meng Y.Q., Jin J., Gao G. (2020). PlantRegMap: Charting functional regulatory maps in plants. Nucleic Acids Res..

[56] Jin J., Tian F., Yang D.C., Meng Y.Q., Kong L., Luo J., Gao G. (2017). PlantTFDB 4.0: Toward a central hub for transcription factors and regulatory interactions in plants. Nucleic Acids Res..

[57] Jin J., Zhang H., Kong L., Gao G., Luo J. (2014). PlantTFDB 3.0: A portal for the functional and evolutionary study of plant transcription factors. Nucleic Acids Res..

[58] Jin J., He K., Tang X., Li Z., Lv L., Zhao Y., Luo J., Gao G. (2015). An *Arabidopsis* transcriptional regulatory map reveals distinct functional and evolutionary features of novel transcription factors. Mol. Biol. Evol..

[59] Babicki S., Arndt D., Marcu A., Liang Y., Grant J.R., Maciejewski A., Wishart D.S. (2016). Heatmapper: Web-enabled heat mapping for all. Nucleic Acids Res..

