# Supplementary material for: De Novo Transcriptome Analysis Reveals Flowering-Related Genes That Potentially Contribute to Flowering-Time Control in the Japanese Cultivated Gentian Gentiana triflora
Source: Int J Mol Sci. 2022 Oct 4;23(19):11754. doi: 10.3390/ijms231911754 (PMC9570441; doi:10.3390/ijms231911754)
Supplement: Supplementary file 1 [file ijms-23-11754-s001.zip › Supplementary_FigsR3.pdf]

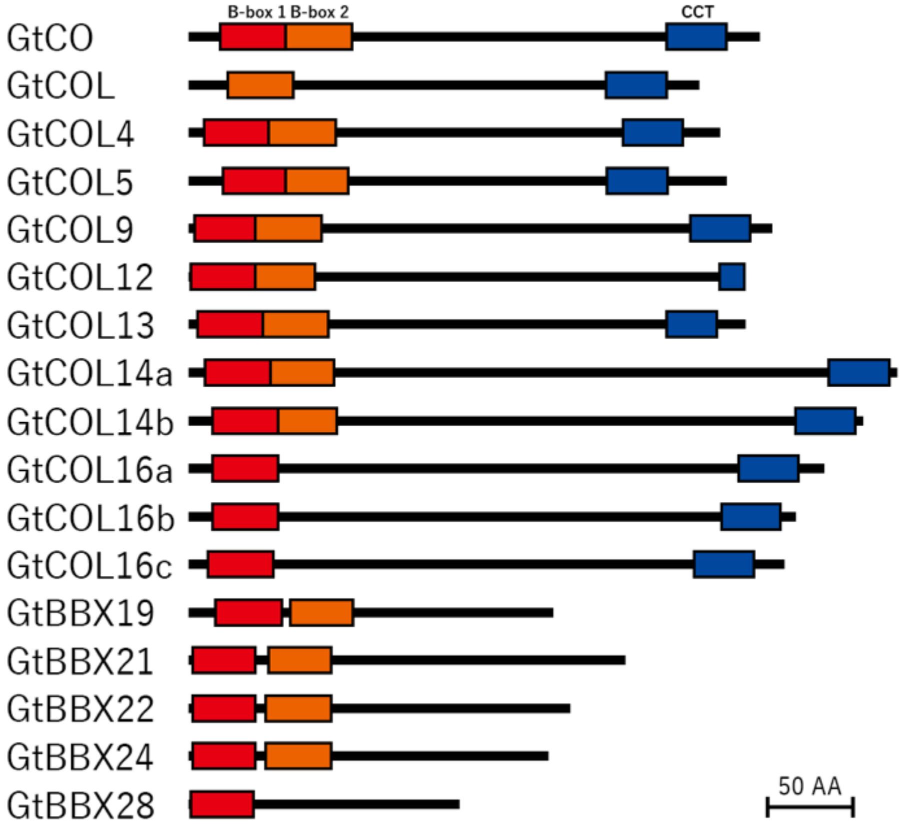

Figure S1. Conserved motifs of the BBX family members in *Gentiana triflora*.

Red, orange, and blue rectangles indicate the B-box1, B-box2, and CCT domains, respectively. GtCOL12 was not a full length. Scale bar represents 50 amino acids.

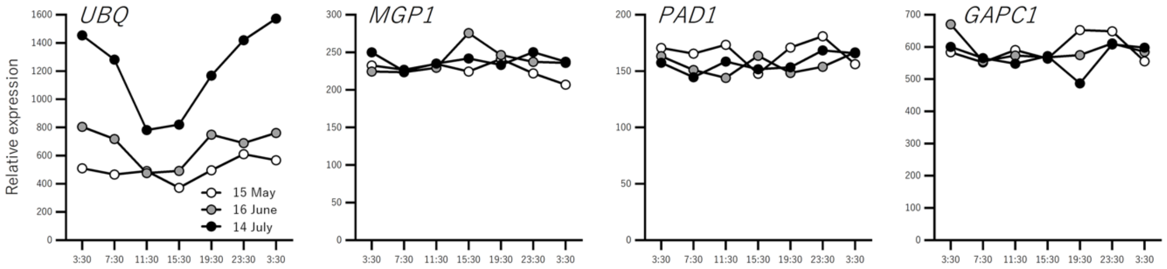

Figure S2. Selected genes as reference genes for quantitative real-time RT-PCR in gentians.

Y-axis shows the TPM value of RNA-seq analysis and X-axis shows time points.

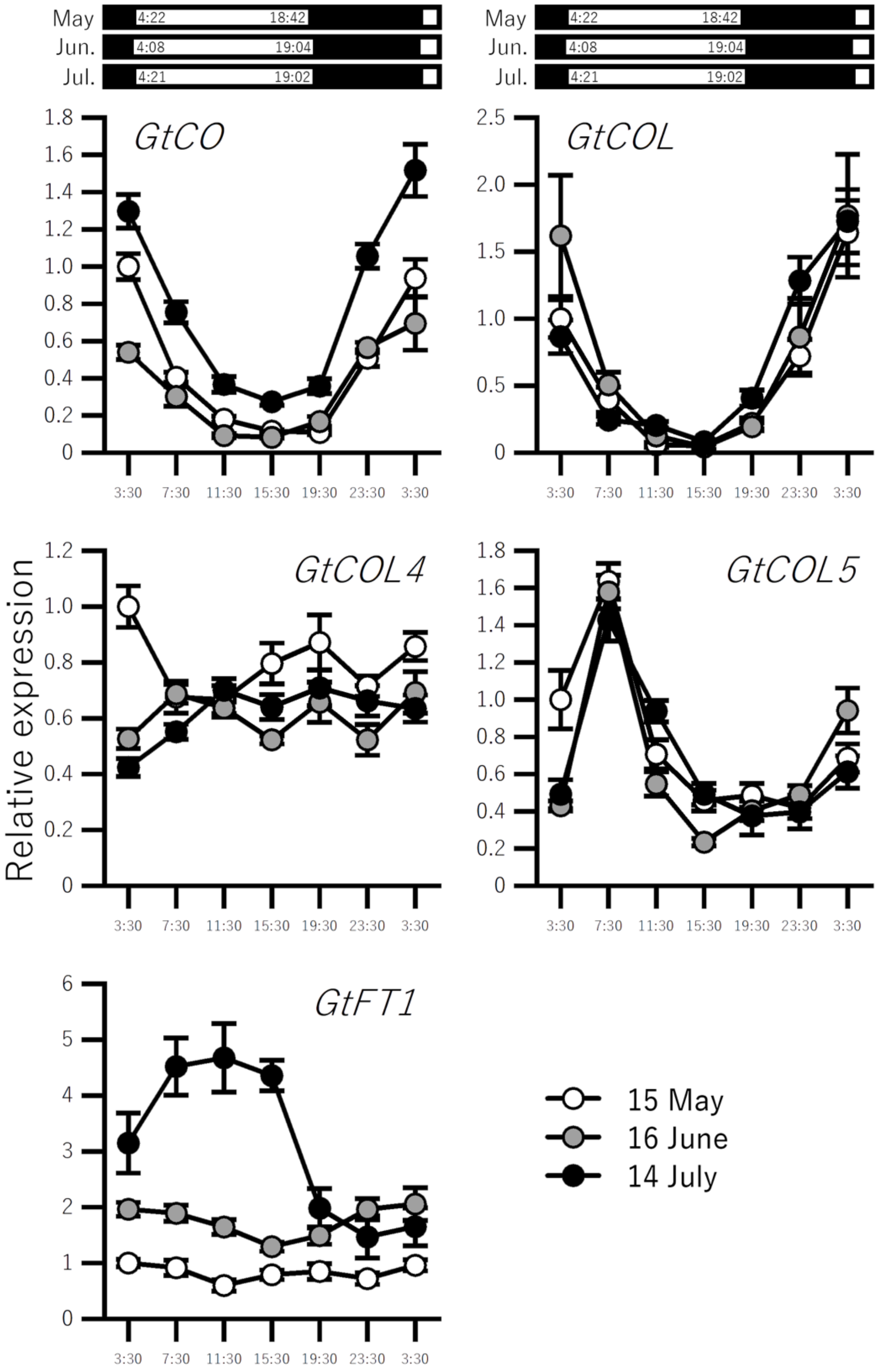

Figure S3. Validation of RNA-seq results using real-time PCR (qRT-PCR) analysis in field-grown gentian plants.

The same RNA samples used in Figure 1A were subjected to this analysis. N = 5–10. White and black boxes indicate day and night period, respectively.

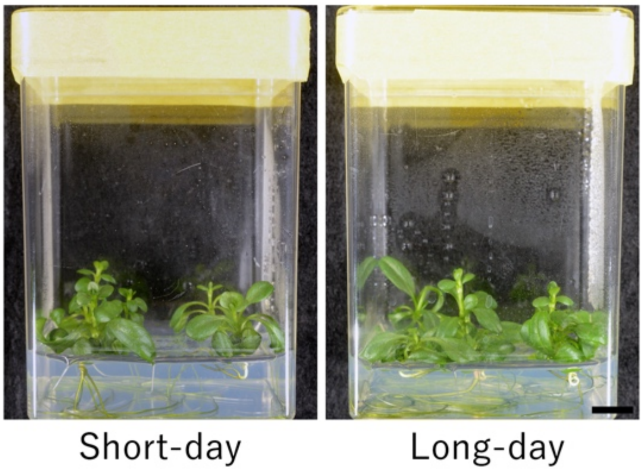

Figure S4. In vitro–grown seedlings prior to sampling.

Seedlings at approximately 3 months after sowing, as presented in Figure 1B. No flower bud was formed. Scale bar = 1 cm.

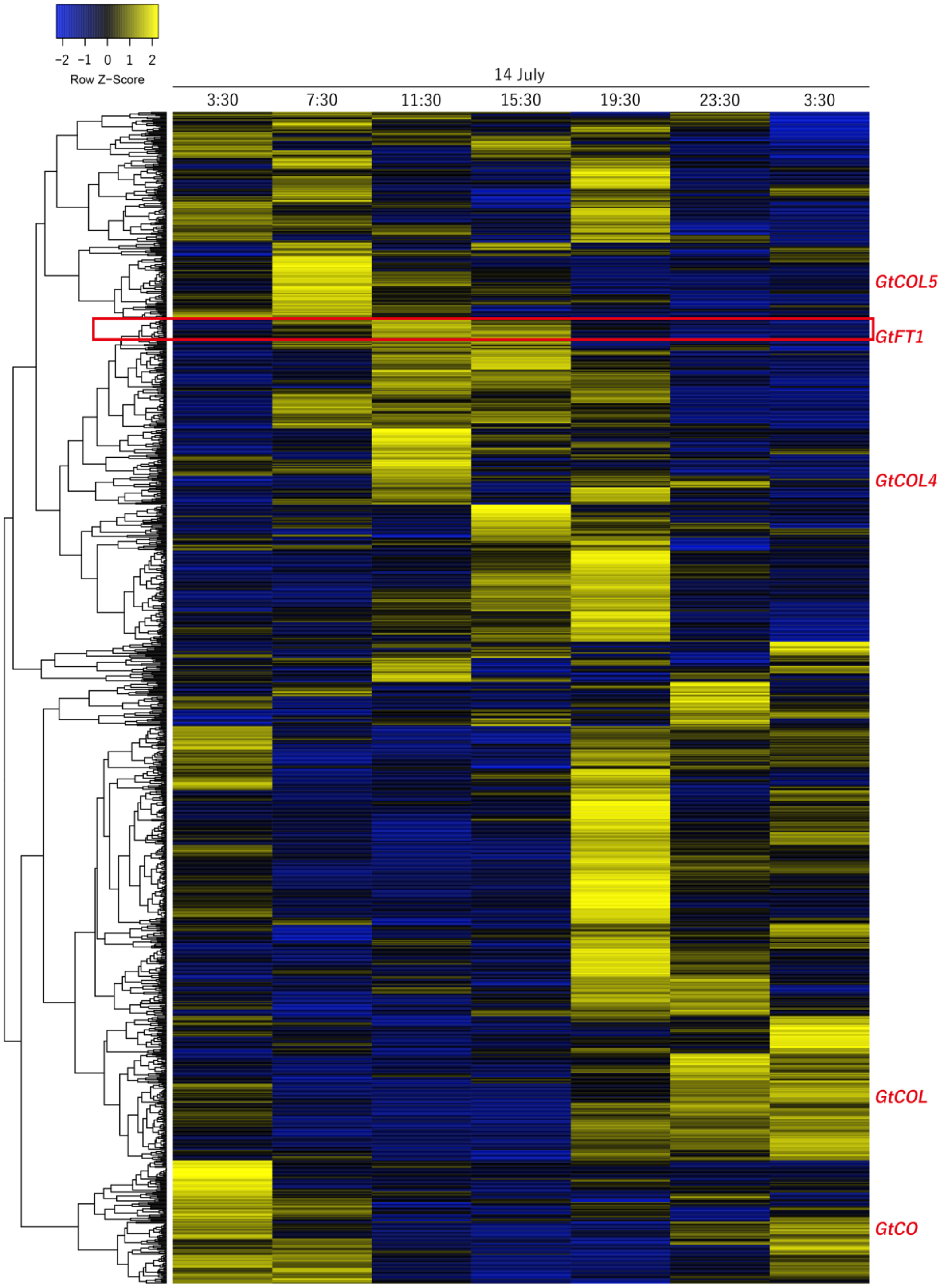

Figure S5. Heatmap and hierarchical clustering of transcription factor gene expression on 14 July.

Selected transcription factor genes used for the coexpression cluster analysis are shown in Table S6. RNA-seq–based heatmap analysis of expression was performed as described in the Materials and Methods. A close-up of the red rectangle is shown in Figure 6A.

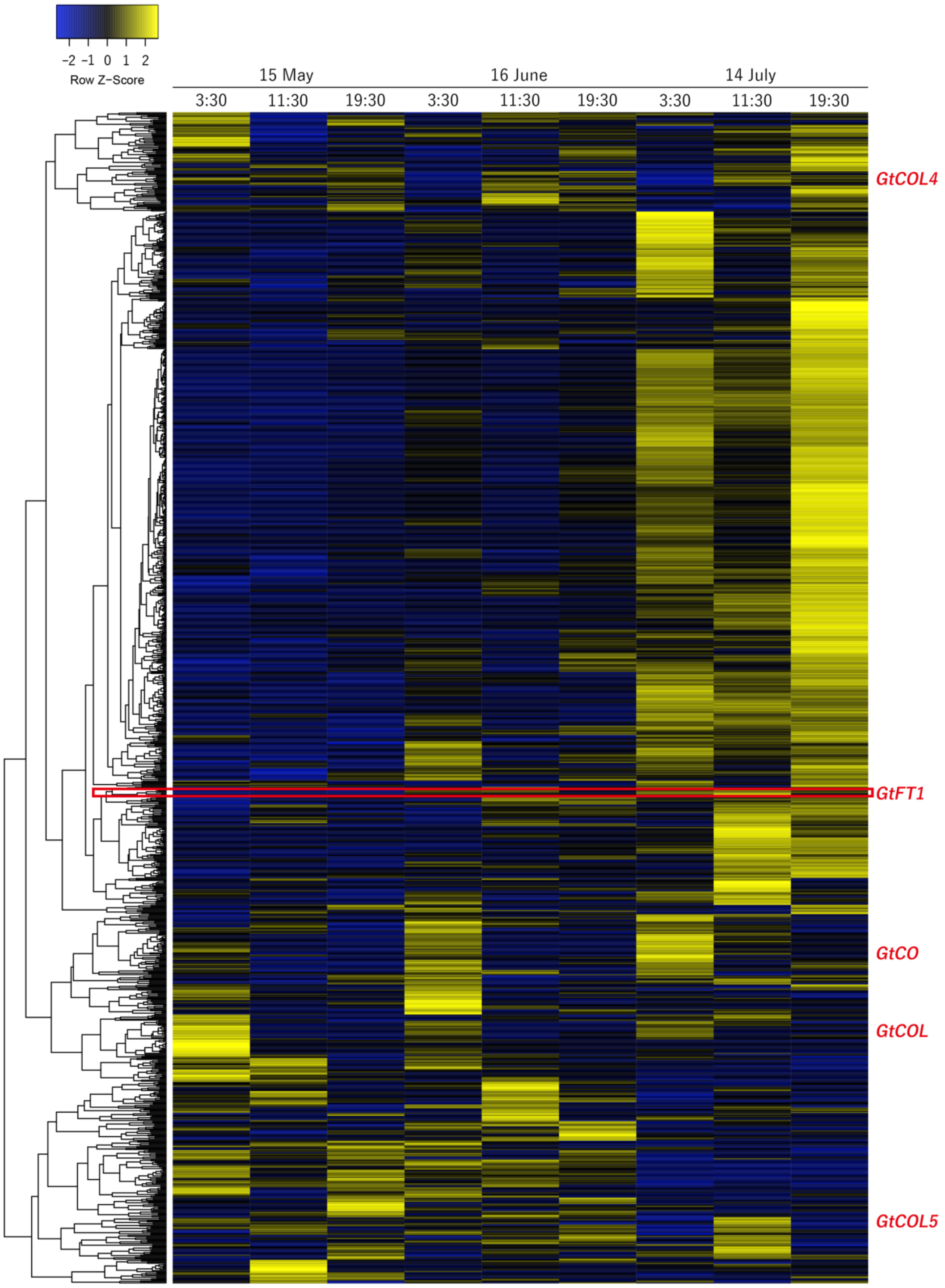

Figure S6. Heatmap and hierarchical clustering of gene expression over three months.

Selected transcription factor genes used for the coexpression cluster analysis are shown in Table S7. RNA-seq–based heatmap analysis of expression was performed as described in the Materials and Methods. A close-up of the red rectangle is shown in Figure 6B.

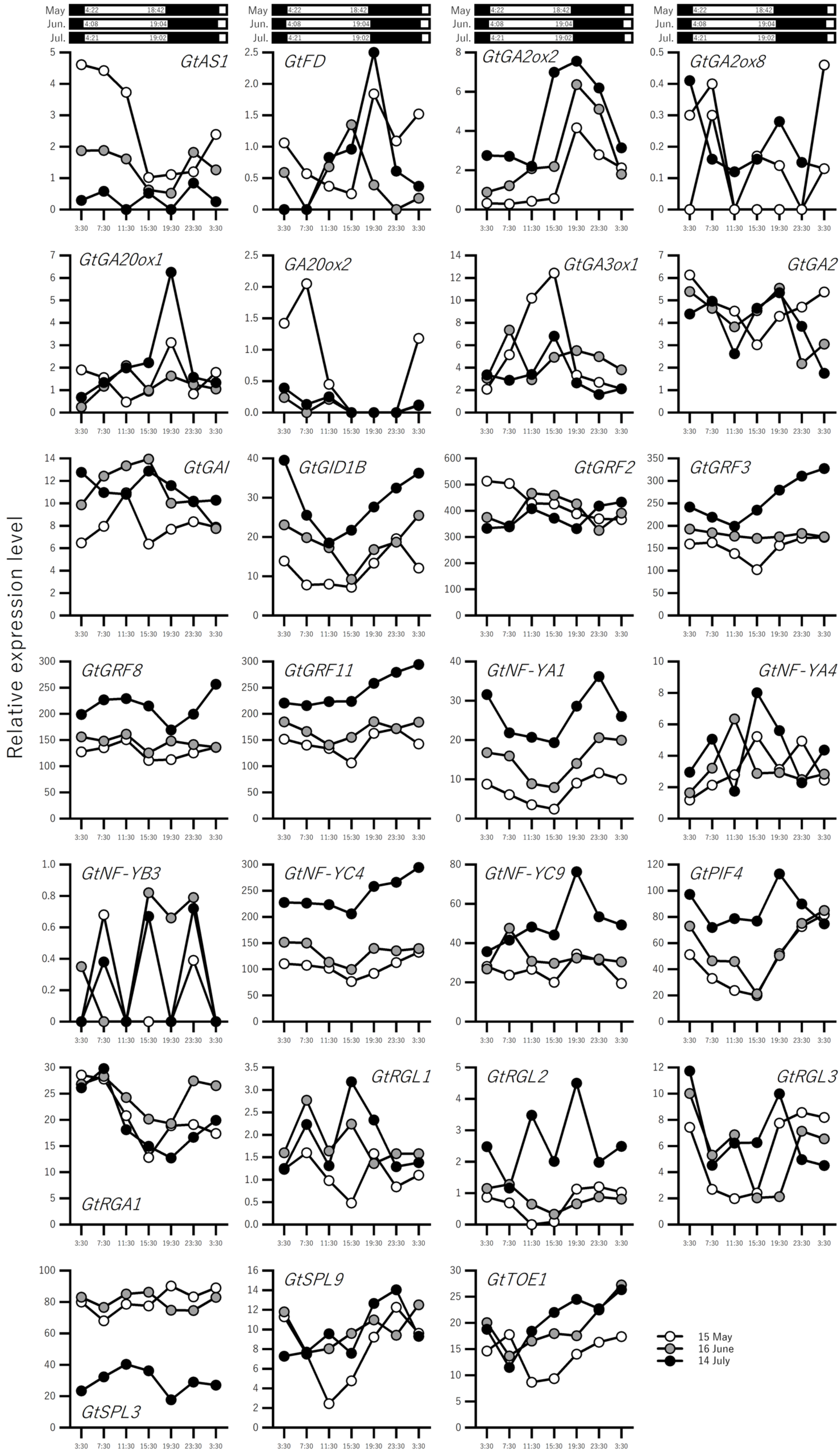

Figure S7. Expression profiles of genes involved in the phytohormone-related pathway in field-grown gentians.

Gentian plants grown under natural conditions in the field were used. The details of sampling are shown in Figure 1A. White and black boxes indicate the day and night periods, respectively. Among 34 genes with hits for the FLOR-ID hormone pathway, 27 genes encoding full-length proteins (except *GtFT1*) are shown.
